# PICOLINIC ACID, A CATABOLITE OF TRYPTOPHAN, HAS AN ANABOLIC EFFECT ON MYOBLASTS AND INCREASES MOBILITY IN C. ELEGANS

**DOI:** 10.1093/geroni/igae098.4249

**Published:** 2024-12-31

**Authors:** Gustavo Duque, Daniel Rivas, Abdelrahman AlOkda, Ivan Baltasar-Fernandez, Jeremy Van Raamsdonk

**Affiliations:** Research Institute of the McGill University Health Centre, Montreal, Quebec, Canada; Research Institute of the McGill University Health Centre, Montreal, Quebec, Canada; Research Institute of the McGill University Health Centre, Montreal, Quebec, Canada; University of Castilla-La Mancha, Toledo, Castilla-La Mancha, Spain; Research Institute of the McGill University Health Centre, Montreal, Quebec, Canada

## Abstract

Osteoporosis, sarcopenia, and osteosarcopenia (simultaneous occurrence of these diseases) are highly prevalent in older persons and associated with falls and fractures. Compounds with dual anabolic effects on muscle and bone could become the treatment of choice for these conditions. We have previously reported the anabolic effect of picolinic acid (PIC) on bone in vitro and in vivo. However, its effect on muscles remains to be elucidated. Therefore, the aim of the study was to examine the effect of PIC on muscle. C2C12 myoblasts were treated with PIC (1, 50 and 100 µM) or vehicle for 7 days. Transcription factors involved in myogenesis were evaluated by western blot, and the fusion index and myotubules’ length were calculated in confocal microscopy images at timed intervals (Day 1, 3, 5 and 7). In addition, C. elegans were treated with 1mM PIC, and the frequency of thrashing (mobility) was evaluated at timed intervals (Day 1, 8 and 16). PIC-treated myoblasts showed a higher and earlier expression of myosin II and MyoD transcription factors (p < 0.001). On day 5, PIC-treated myotubes were significantly longer when treated with 50 µM (+80.7 µm, p=0.001) and 100 µM (+73.0 µm, p=0.002) than vehicle-treated controls. The frequency of thrashing was also significantly increased in the PIC-treated C. elegans at all timed intervals (p < 0.001). In conclusion, this study demonstrates that, in addition to its anabolic effect on bone, PIC also has an anabolic effect on muscle, which opens up exciting possibilities for further exploration in animal models and future geroscience research.

